# Genome-Wide Identification of bZIP Transcription Factor Genes and Functional Analyses of Two Members in *Cytospora chrysosperma*

**DOI:** 10.3390/jof8010034

**Published:** 2021-12-30

**Authors:** Dasen Wen, Lu Yu, Dianguang Xiong, Chengming Tian

**Affiliations:** 1 The Key Laboratory for Silviculture and Conservation of Ministry of Education, College of Forestry, Beijing Forestry University, Beijing 100083, China; Dasen_Wen@163.com (D.W.); luciaciacia_yu@163.com (L.Y.); 2 Beijing Key Laboratory for Forest Pest Control, College of Forestry, Beijing Forestry University, Beijing 100083, China

**Keywords:** *Cytospora chrysosperma*, bZIP transcription factor, fungal growth, stress response, pathogenicity

## Abstract

The basic leucine zipper (bZIP) transcription factor (TF) family, one of the largest and the most diverse TF families, is widely distributed across the eukaryotes. It has been described that the bZIP TFs play diverse roles in development, nutrient utilization, and various stress responses in fungi. However, little is known of the bZIP members in *Cytospora chrysosperma*, a notorious plant pathogenic fungus, which causes canker disease on over 80 woody plant species. In this study, 26 bZIP genes were systematically identified in the genome of *C. chrysosperma*, and two of them (named *CcbZIP05* and *CcbZIP23*) significantly down-regulated in *CcPmk1* deletion mutant (a pathogenicity-related mitogen-activated protein kinase) were selected for further analysis. Deletion of *CcbZIP05* or *CcbZIP23* displayed a dramatic reduction in fungal growth but showed increased hypha branching and resistance to cell wall inhibitors and abiotic stresses. The *CcbZIP05* deletion mutants but not *CcbZIP23* deletion mutants were more sensitive to the hydrogen peroxide compared to the wild-type and complemented strains. Additionally, the *CcbZIP23* deletion mutants produced few pycnidia but more pigment. Remarkably, both *CcbZIP05* and *CcbZIP23* deletion mutants were significantly reduced in fungal virulence. Further analysis showed that *CcbZIP05* and *CcbZIP23* could regulate the expression of putative effector genes and chitin synthesis-related genes. Taken together, our results suggest that *CcbZIP05* and *CcbZIP23* play important roles in fungal growth, abiotic stresses response, and pathogenicity, which will provide comprehensive information on the *CcbZIP* genes and lay the foundation for further research on the bZIP members in *C. chrysosperma*.

## 1. Introduction

Transcription factors (TFs) are involved in all kinds of biological processes, such as cellular growth, differentiation, and the response to environmental factors, through modulating the expression of downstream genes. Their roles, including specific promoter sequences binding to transcriptional inhibition or activation, interaction with other TFs or molecular chaperones, as well as post-translational modifications, are crucial to regulating the transcription of target genes [1,2,3,4]. According to the similarities of primary and/or three-dimensional structure of the DNA-binding and multimerization domains, TFs can be categorized into different families, for example, the basic leucine zipper (bZIP) proteins, fungal-specific Zn2Cys6 proteins, Cys2His2 (C_2_H_2_) zinc finger proteins, MADS-box proteins, helix-loop-helix (HLH) proteins, homeobox proteins, and so on [5,6,7]. Among them, the bZIP proteins, widely distributed in eukaryotes, are regarded as one of the central regulators functioning in various biological processes in pathogenic fungi, such as fungal development, stress responses, and pathogenicity [8,9,10]. The bZIP domain is the typical feature of these proteins, which is generally 60–80 amino acids in length and consists of two functionally distinct parts: a highly conserved basic region and a variable leucine-zipper region, hence called bZIP [11,12,13]. The basic region is responsible for DNA binding and nuclear import with a nuclear localization signal and an invariant motif of N-X_7_-R/K, which generally connects to the first heptad repeat of the leucine zipper by a short hinge region consisting of nine amino acid residues [14]. The leucine zipper region is an amphipathic sequence and mediates homo- and heterodimerization, contains variable length with a leucine every seven amino acids or other bulky hydrophobic amino acids and forms a coiled-coil structure [15,16,17,18]. In addition to the bZIP domain, several other DNA-binding domains, such as glutamate-rich, proline-rich, and acidic domains, have been identified in the bZIP proteins, which are also involved in the transactivation function [19,20,21].

With the increasing availability of genome data, numerous bZIP members have been identified and functionally characterized in various fungi. Intriguingly, a different number of bZIP genes were found in different fungal species. Previous studies identified 22 bZIP members in *Magnaporthe oryzae*. Some of them are involved in fungal pathogenicity, and several of them contribute to the stress response [22]. In *Coniothyrium minitans*, 34 bZIP members have been identified, all of which are involved in different stages of mycoparasitism [23]. In addition, there are 26 bZIP members in *Fusarium graminearum* [24] and 28 bZIP members in *Ustilaginoidea virens* [25]. Remarkably, the bZIP proteins show pleiotropic roles in plant pathogenic fungi, such as fungal development, nutrient utilization, environmental stress (oxidative, osmotic, cell wall integrity inhibitors) responses, virulence, unfolded protein response (UPR), control of primary and secondary metabolism, etc. [26,27,28,29,30,31]. For example, YAP1 is critical for the oxidative stress response [32]. HapX mediates iron balance, which is important for fungal virulence [33]. Atf1 regulates the transcription of the laccase and peroxidase-encoding genes and can assist the fungus to overcome reactive oxygen species (ROS)-mediated plant defenses [34]. Hac1 contributes to the unfolded protein response [35]. MeaB affects the synthesis of nitrogen metabolites [36]. FlbB is necessary for gliotoxin production and asexual reproduction [37]. However, many studies also show that single deletion of some bZIP genes does not display detectable defects compared to the wild-type, indicating the putative functional redundancy among this family [38].

*Cytospora chrysosperma* (Pers.) Fr, a notorious pathogenic fungus, attacks plenty of woody plants including poplar (*Populus* spp.) and causes destructive canker disease [39,40,41], resulting in serious forestry economic and ecological damage every year around the world, especially in China [42]. The fungus overwinters with mycelia and conidia in the diseased or dead plant, which then act as primary infection sources and infect the host plant through wounds [43]. As a necrotrophic fungus, akin to the *Cryphonectria parasitica* and *Valsa mali*, the colonization of *C. chrysosperma* is dependent on the conditions of host trees, which will initially be restricted to injured or dying bark in the healthy polar. Once the tree vigor becomes weak, for example, stems weakened by drought or freeze damage, *C. chrysosperma* will rapidly and expansively spread, degrade the plant cells, and quickly kill the host plants. Finally, the infected plants will exhibit obvious visible symptoms, such as collapsed rotting stems, the formation of ring-like spots, or even the death of the entire tree [44,45]. However, effective disease prevention and control measures are still limited. Clarification of the pathogenesis is important to develop durable and efficient control strategies for plant diseases. Recently, several pathogenicity-related genes have been identified and functionally analyzed in *C. chrysosperma* [46,47,48]. Among them, the pathogenicity-related mitogen-activated protein kinase *CcPmk1*, acting as a global regulator, modulates the expression of downstream TFs and can be used as a potential target for broad-spectrum plant disease resistance [47]. The downstream fungal-specific transcription regulator, *CcSge1*, belonging to the Gti1/Pac2 family, is required for the expression of effector genes and pathogenicity [49]. Intriguingly, *CcCAP1*, one of the putative effector genes regulated by *CcSge1*, belonging to the CAP superfamily (PF00188), can inhibit the plant immunity and promote the infection on host poplar [50]. Therefore, the TFs are the essential components to connect the upstream signals and downstream effectors, which can be regarded as the potential targets to reveal the molecular pathogenic mechanism of plant pathogens. However, little is known about the functions of TFs in *C. chrysosperma*.

In this study, we identified and characterized 26 bZIP genes in *C. chrysosperma*, namely, *CcbZIP01* to *CcbZIP26*. Among them, *CcbZIP05* and *CcbZIP23*, significantly down-regulated in *CcPmk1* deletion mutant, were selected for further analyses. The results showed that both *CcbZIP05* and *CcbZIP23* contribute to the fungal development, stress response, and pathogenicity of *C. chrysosperma*. These results provide a foundation for further study of other bZIP members in *C. chrysosperma.*

## 2. Materials and Methods

### 2.1. Identification of bZIP Transcription Factors in C. chrysosperma

The draft genome sequence of *C. chrysosperma* was sequenced by our lab. (Unpublished data, NCBI GenBank accession number JAEQMF000000000.) The bZIP protein sequences of various fungal species were downloaded from NCBI (https://www.ncbi.nlm.nih.gov/, accessed on 11 October 2019) or FTFD (http://ftfd.snu.ac.kr/index.php?a=view, accessed on 24 December 2019) and were used as queries for BLASTP searches (E-value cutoff < 1 × 10^−5^) against the *C. chrysosperma* genome. The resulting bZIP domains were used to generate motif by using the hidden Markov model (HMM) with E-value threshold ≤ 500, and then searched in the *C. chrysosperma* genome [51]. All output genes (removing the repetitive sequences) manually confirmed the existence of the bZIP domain (InterPro ID #IPR004827) by using the InterProScan (http://www.ebi.ac.uk/interpro/, accessed on 26 December 2019). The sequences containing the bZIP domain were regarded as candidate *CcbZIPs* for the further analyses.

### 2.2. Gene Structural Characterization and Phylogenetic Analysis

The theoretical isoelectric points and molecular weights of the identified gene products were predicted by using the ExPASy proteomics server (http://web.expasy.org/protparam/, accessed on 30 December 2019). The genome sequences of the bZIP genes and the corresponding CDS sequences were committed to the Gene Structure Display Server (GSDS, http://gsds.cbi.pku.edu.cn/, accessed on 27 December 2020) to analyze the number and alignment of introns and exons.

Multiple sequence alignments of the identified bZIP TF sequences were performed using MEGA6 [52], followed by minor modifications using GeneDoc [53]. A phylogenetic analysis was performed with raxmlGUI1.3 software [54], using the maximum likelihood method, and a bootstrap test with 1000 iterations. TBtools (V0.66836) was used to compile phylogenetic trees and relative expression level in heatmap [55]. The Pathogen Host Interaction Base (PHIB) (http://phi-blast.phi-base.org/, accessed on 24 December 2020) was used to identify bZIP homologs that had been functionally analyzed.

### 2.3. Conditions for Fungal Strains’ Growth and Treatment

The wild-type (WT) strain (CFCC 89981) of *C. chrysosperma* was derived from the Forest Pathology Laboratory of Beijing Forestry University (Strain No: G-YS-11-C1) [56]. All strains were cultured on potato dextrose agar (PDA, 200 g potato, 20 g dextrose, 15 g agar, and 1 L water) medium at 25 °C. Potato dextrose broth (PDB, 200 g potato, 20 g dextrose, and 1 L water) was used to culture mycelium ready for DNA and RNA extraction. To analyze the differences in vegetative growth among the strains, growth patterns were monitored and colony diameters were measured daily post-inoculation (dpi) on PDA plates and continued for 3 days. To calculate the conidial production, the number of pycnidia was counted at 30 dpi. To assess the stress response, small agar blocks were cut from the edges of 2-day-old cultures and placed on PDA plates supplemented with different stress agents including 0.05 M NaCl, 0.04 M KCl, 0.8 M Sorbitol, 20 μg/mL calcofluor white (CFW), 200 μg/mL Congo red (CR), and 0.01% Sodium dodecyl sulfate (SDS), then incubated at 25 °C in the dark for 60 h. In the oxidative stress test, a 1 × 10^5^ spores/mL suspension was added into the melted PDA medium (about 45~50 °C) and then poured into 9-cm plates with a drop of 5 M or 10 M H_2_O_2_ on a filter in the center of the medium plate. The diameter of the inhibition zone was measured at 60 hpi. In order to visualize the chitin deposition and distribution, conidia of each strain were inoculated on 1-mm PDA slides for 1 day and then stained with 10 mg/mL CFW. All tests were repeated three times, and all data were analyzed in SPSS 16.0 by One-Way ANOVA and Duncan’s range test to measure specific differences between strains. Significant changes between different treatments were calculated with *p* < 0.05.

### 2.4. Generation of CcbZIP Gene Deletion Mutants

The *CcbZIP* genes were disrupted using the split-marker system. In the case of *CcbZIP05*, firstly, the upstream (~1.3 kb) and downstream (~1.2 kb) flanking sequences of *CcbZIP05* were PCR amplified with specific primer pairs CcbZIP05-5Ffor/rev and CcbZIP05-3Ffor/rev, as shown in the Table S1. Then, the hygromycin B resistance cassette (HPH), which includes sequences of approximately ~20 bp that overlap with the 5′ and 3′ flanking sequences, was amplified by the specific primer pairs CcbZIP05-5Frev+/HY-R and YG-F/CcbZIP05-3Ffor+. Then, the upstream and downstream fragments and two-thirds of the hygromycin cassette were fused by overlapping PCR using the primers CcbZIP05-5Ffor/HY-R and YG-F/CcbZIP05-3Frev, respectively. The resulting two overlapped fragments were transformed into the protoplasts of *C. chrysosperma* by using the PEG-mediated transformation, as described previously [57]. Finally, the gene deletion mutants were screened by PCR with the primer pairs External-CcbZIP05for/rev and Internal-CcbZIP05for/rev, then confirmed by Southern blotting analysis according to the manufacturer’s instructions (DIG-High Prime DNA Labeling and Detection Starter Kit I).

To complement the *CcbZIP05* deletion mutant, fragments containing upstream ~1.5 kb of local promoter sequence, open reading frame, and downstream ~0.2 kb of the sequence were cloned from gDNA using primer CcbZIP05-Compfor/Comprev. The obtained PCR products and the geneticin-resistant cassette were co-transformed into the protoplasts of the deletion mutant strain. Successful complementation was confirmed by PCR with the primer pair External-CcbZIP05for/rev and Internal-CcbZIP05for/rev.

The *CcbZIP23* deletion mutants were obtained in the same way and all the primers mentioned are listed in Table S1.

### 2.5. Pathogenicity Tests

For the pathogenicity test, healthy annual twigs of *Populus euramericana* (a susceptible *Populus* species) were used. *C. chrysosperma* initiated infection through wounds; therefore, the selected branches were scalded with a 5-mm diameter hot iron rod and then inoculated with 5-mm agar plugs taken from the leading edge of colonies of wild-type, gene deletion mutant, and complementary strains, respectively. The inoculated twigs were placed on top of trays, kept humid with distilled water, and incubated at 25 °C in a day/night cycle. The lesions of the inoculated twigs were photographed and measured at 3 dpi and 6 dpi, respectively. The experiments were repeated three times, with at least 25 twigs for each strain.

### 2.6. RNA Extraction and RT-qPCR Analysis

To analyze the expression levels of *CcbZIP05* and *CcbZIP23* in *CcPmk1*-deletion mutants, 5-mm agar plugs of wild-type, Δ*CcPmk1*, and complemented strains were added into PDB supplemented with sterilized poplar twigs, respectively, and then incubated at 25 °C with shaking at 150 rpm for 2 days. To assess the expression levels of candidate effector genes in Δ*CcbZIP05* and Δ*CcbZIP23* mutants, the same method was used. To examine the expression of putative chitin synthase-related genes (CHS), the strains were growth in PDB at 25 °C with shaking at 150 rpm for 3 days.

Total RNA was extracted with TRIzol reagent (Invitrogen, USA) according to the manufacturer’s instructions. The total RNA was treated with DNase before reverse transcription. Agarose gel electrophoresis was used to examine the quality of total RNA and to estimate its concentration. First-strand cDNA was synthesized from 1 μg RNA with ABScript II cDNA Fist-Strand Synthesis Kit (ABclonal, China), according to the manufacturer’s instructions. The qRT-PCR assay was conducted with 2X Universal SYBR Green Fast qPCR Mix (ABclonal, China) using an ABI 7500 real-time PCR system (Applied Biosystems, USA). The *CcActin* gene served as the endogenous control for all qRT-PCR analyses. All samples were independently subjected to three replicate experiments. The relative expression of genes was calculated by using the 2^−ΔΔCt^ method.

## 3. Results

### 3.1. Identification of bZIP Genes in C. chrysosperma

To characterize the bZIP members of *C. chrysosperma*, we downloaded the bZIP protein sequences of eight fungal species, including 22 bZIPs in *M. oryzae*, 15 bZIPs in *Botrytis cinerea*, 20 bZIPs in *Verticillium dahliae*, 15 bZIPs in *Saccharomyces cerevisiae*, 25 bZIPs in *Colletotrichum gloeosporioides*, 17 bZIPs in *Sclerotinia sclerotiorum*, 15 bZIPs in *Cryphonectria parasitica*, and 26 bZIPs in *F. graminearum*. Then we extracted the bZIP domain sequences of these 155 bZIPs and used them as templates to search through the *C. chrysosperma* genome database with HMMER, and 56 putative hits were obtained. Subsequently, the acquired 56 sequences were manually confirmed for the presence of bZIP domain by using InterProScan on the EBI web server. Finally, 26 putative sequences were identified, which contained the bZIP domain including bZIP_1 domain (PF00170), bZIP_Maf (PF03131), or bZIP_2 (PF07716). These genes were named *CcbZIP01* to *CcbZIP26* according to their locus order. The length of these 26 CcbZIP proteins ranged from 163 to 633 aa, which contained 0 to 3 introns, and theoretical molecular weight ranged from 18.24 to 69.87 KDa, with predicted pI values ranging from 4.635 to 11.082. Among them, the largest difference in length between the cDNA and gDNA sequences of *Ccbzip04* was 1709 base pairs (bp) (Table 1).

### 3.2. Analysis of Conserved Domains and Motif in CcbZIP Proteins

**Figure 1:**
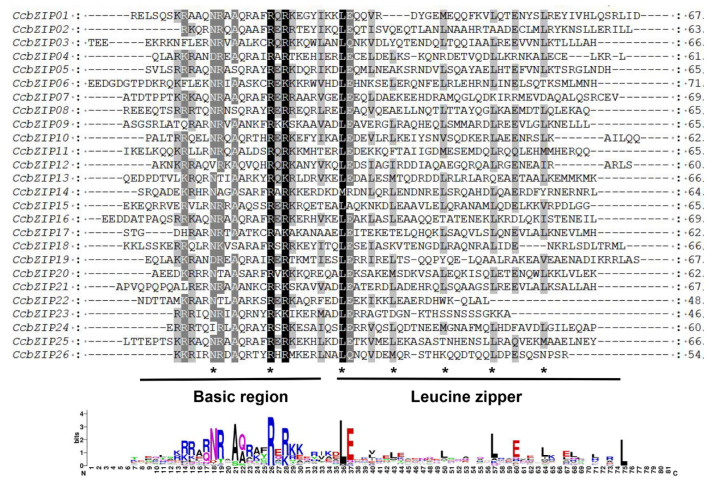
Multiple sequence alignment of bZIP domain in *CcbZIP* genes. Amino acid sequences of bZIP domain were aligned by MUSCLE V3.8.1551 with minor modifications. Black, medium gray, and light gray indicate the conserved percent of 100%, >80%, and >60%, respectively. The short, black lines in the middle represent a typical bZIP domain; asterisks show the conserved amino acids of bZIP domains. The bottom part is the sequence logo formed by bZIP domain from *CcbZIP* genes, and the larger letters of the amino acid residues, the more frequently they appear at the same site.

The classical bZIP domain usually consists of a basic region and a leucine-zipper region. The basic region is highly conserved, with an N-X_7_-R/K motif and the variable leucine-zipper region with a leucine or other hydrophobic amino acids every seven amino acids. To investigate the characteristics of the *CcbZIP* domain, multiple amino acid sequences of the bZIP domain were aligned, as shown in Figure 1. The core asparagine (N) residues of the bZIP domains of *CcbZIP04* and *CcbZIP19* were substituted with aspartic acid (D), while they were substituted with valine (V) in *CcbZIP12* and isoleucine (I) in *CcbZIP24*. Except for the four CcbZIPs mentioned above, all the remaining *CcbZIP* domains contained invariant N-X_7_-R motif. The first heptad repeat Leu was highly conserved in the leucine zipper region, except for *CcbZIP14,* which was replaced by Met. However, the following heptad repeat Leu was often replaced by other bulky hydrophobic amino acids, e.g., the second heptad repeat Leu in *CcbZIP*13, -20, and -26 was replaced by Met.

In addition, three *CcbZIP* members contained other predicted domains except for the bZIP domain, which may have different specific functions. *CcbZIP03* contained an Aft1_HRA domain (IPR021755), Aft1_OSM domain (IPR020956), and Aft1_HRR domain (IPR021756) at the N-terminal of the sequence. *CcbZIP07* contained a Hap4_TF_heteromerisation domain (IPR018287) at the N-terminal of the sequence. *CcbZIP25* contained a PAP1 domain (IPR013910) at the C-terminal of the sequence (Figure S1).

### 3.3. Intron Numbers and Their Distribution Pattern in CcbZIPs

**Figure 2:**
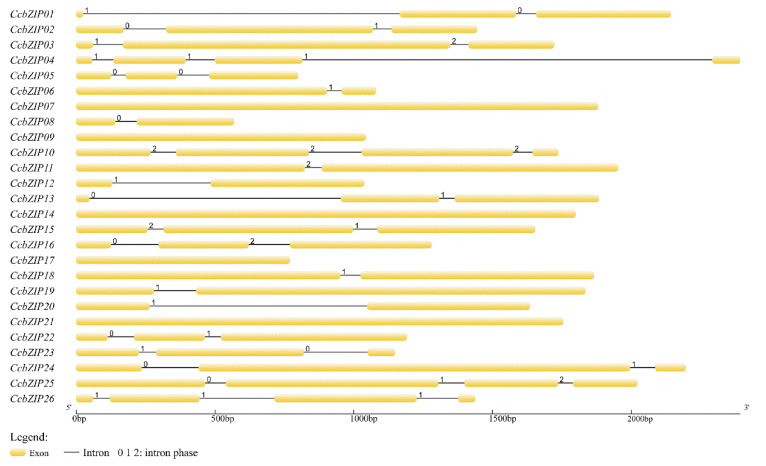
The gene structures of *CcbZIPs*. Arrangement of introns and exons were displayed by GSDS version 2.0. Exons are drawn to scale in the yellow boxes. The black lines connecting the two exons represent introns. The numbers 0, 1, and 2 represent the splicing phase of intron. Phase 0 means intron splicing site is between codons, while phase 1 and phase 2 mean the intron splicing site is located after the first and second nucleotide of a codon, respectively.

The evolutionary imprint of some gene families can be revealed by the intron/exon arrangement [58]. To further understand the structural characteristics of the *CcbZIP* gene, the intron arrangement pattern and insertion sites of *CcbZIPs* were identified using the GSDS. The different intron distribution patterns are displayed in Figure 2. Among them, 7 *CcbZIP* genes contained one intron, 11 *CcbZIP* genes contained two introns, and 3 *CcbZIP* genes contained three introns. The intron lengths ranged from 52 to 1477 bp; 58% of the introns were less than 100 bp and only two introns were larger than 1000 bp.

**Figure 3:**
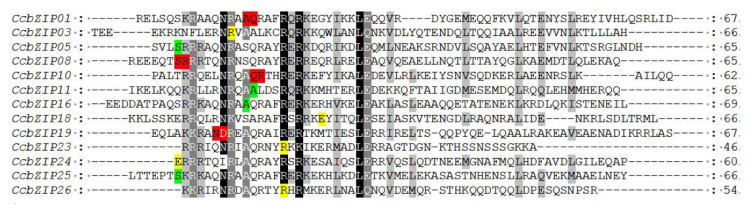
Analysis of splicing phase of intron in the bZIP domains. The yellow letters and the green letters mean splicing sites are located after the first and second nucleotide of a codon, respectively. The red letter means splicing sites are located between two codons. Black, medium gray, and light gray indicate the conserved percent of 100%, >80%, and >60%, respectively.

Previous reports have shown that the insertion position of introns affects gene expression [59]. Since the basic region holds a special status in the bZIP domain, which may bind to specific DNA sequences to regulate gene expression, the distribution pattern of introns within the basic region is important for its functions. We analyzed the pattern of intron positions within the basic, hinge, and leucine regions, and 13 *CcbZIP* genes contained one intron in the bZIP domain region. More than half of the introns’ (*CcbZIP01, -03, -10, -11, -16, -23, -26*) insertion sites were located in the N-X_7_-R motif (Figure 3). Intriguingly, the intron insertion sites of *CcbZIP23* and *CcbZIP26* were located in the core arginine (R) in the N-X_7_-R motif, which are both inserted between the second and third codon.

### 3.4. Phylogenetic Analysis of bZIP Gene Family

To investigate the evolutionary relationship of the *CcbZIP* genes with other fungal bZIP genes, a phylogenetic analysis of bZIP genes in nine fungal species was performed. A phylogenetic tree was produced from 181 fungal bZIP sequences (including the 26 CcbZIP proteins in *C. chrysosperma*) (Table S2), and the defined clades consisting of homologous members are listed in Figure S2, respectively. These 181 bZIPs were divided into eight clades and were assigned to A through H. Overall, the 26 CcbZIP genes were evenly distributed in all these clades, and *CcbZIP23* and *CcbZIP26* belonged to the same subcluster in the clade D. The bZIP superfamily has been reported to have accumulated from a single eukaryotic gene ancestor and has undergone multiple independent amplifications [60]. None of the bZIPs from *C. gloeosporioides* and *S. sclerotiorum* was distributed in clade E, indicating the late divergence of those bZIP genes in these fungi.

Meanwhile, we identified the pathogenicity functions of the respective homologs of the 26 *CcbZIP* genes in *C. chrysosperma* using the online website PHIB (Table S3). The results showed that 11 of *CcbZIP* homologous genes were required for the virulence in different fungi, and one of them (*MoAP1* from *M. oryzae, CcbZIP2*5 homologous) was indispensable for the fungal pathogenicity.

### 3.5. Expression Patterns of CcbZIPs during the Initial Infection Stages

**Figure 4:**
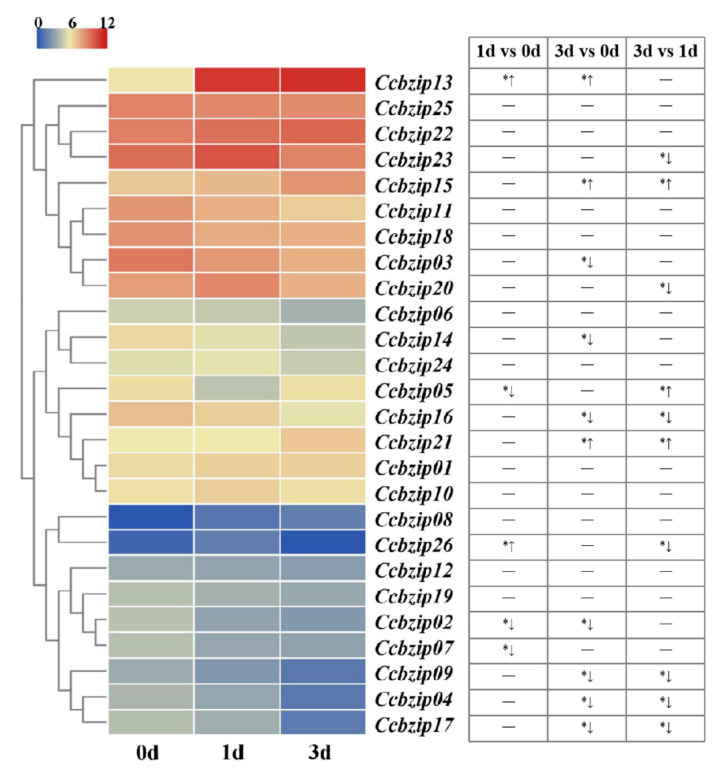
The expression patterns of *CcbZIPs* at the initial infection stages. The heatmap shows the expression data of each *CcbZIPs*. The original FPKM values of *CcbZIP* genes were transformed by log2. The color scale ranging from blue to red indicates the increased expression levels. The differentially expressed genes (|log2foldchange| ≥ 1, *p* < 0.05) are indicated with *. The up arrows represent significantly up-regulated, while the down arrows represent significantly down-regulated.

In order to screen the *CcbZIPs* that might be involved in infection processes, we collected the expression data of 26 *CcbZIP* genes from our recent transcriptome data during the initial infection process (1 dpi and 3 dpi) of *C. chrysosperma* in poplar branches (Table S4), which were submitted to the GEO database with the accession numbers: GEOGSM4959007 (0d-1), GSM4959008 (0d-2), GSM4959009 (0d-3), GSM4959010 (1d-1), GSM4959011 (1d-2), GSM4959012 (1d-3), GSM4959013 (3d-1), GSM4959014 (3d-2), and GSM4959015 (3d-3), respectively [61]. As shown in the Figure 4, the expression levels of the five *bZIP* genes were significantly different at 1 dpi compared to the 0 dpi, including three down-regulated (*CcbZIP02*, *-05*, *-07*) and two up-regulated (*CcbZIP13*, *-26*) *bZIP* genes. Additionally, 10 *bZIP* genes were differentially expressed at the 3 dpi compared to that at 0 dpi, including seven down-regulated (*CcbZIP02, -03, -04, -09, -14, -16, 17*) and three up-regulated (*CcbZIP13*, *-15*, *-21*) *bZIP* genes. Further analysis showed that 10 *CcbZIP* genes were differentially expressed between 3 dpi and 1 dpi. Interestingly, the expression level of *CcbZIP05* was significantly down-regulated at 1 dpi, but basically recovered its expression at 3 dpi. These results suggest that CcbZIP transcription factors may play important roles in fungal pathogenicity of *C. chrysosperma*.

### 3.6. Construction of CcbZIP Deletion Mutants

**Figure 5:**
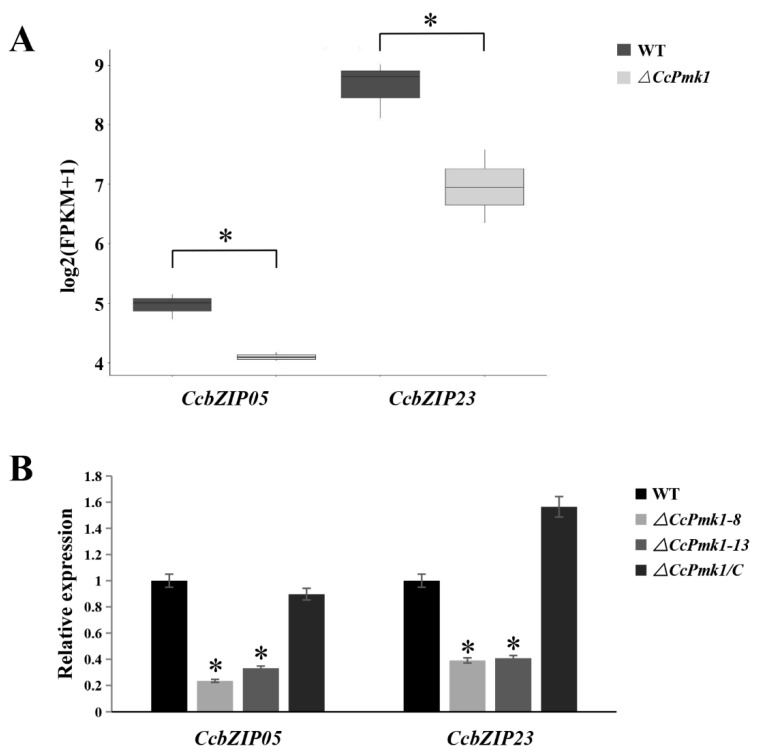
Expression of *CcbZIP05* and *CcbZIP23* in wild-type and *CcPmk1*-deficient mutants. (**A**) The expression patterns of *CcbZIP05* and *CcbZIP23* in Δ*CcPmk1* deletion mutant and wild-type according to the RNA-Seq data. The original FPKM values of *CcbZIP05* and *CcbZIP23* were transformed by log2. (**B**) The qRT-PCR was used to measure the relative expression levels of *CcbZIP05* and *CcbZIP23* in the wild-type, Δ*CcPmk1* mutants, and complemented strains. The *CcActin* gene was used as the reference gene. The experiments were repeated three times. The data were analyzed using Duncan’s range test. The asterisks indicate significant differences (*p* < 0.05).

Mitogen-activated protein kinase (MAPK) signaling cascades are highly conserved in eukaryotes and play essential roles in developmental processes and various cellular responses [62]. Our previous works revealed that the MAKP gene *CcPmk1* plays crucial roles in fungal virulence in *C. chrysosperma* through regulating the expression of downstream genes [48]. Thus, we queried the expression data of the 26 *CcbZIP* genes through the transcriptomic data of *CcPmk1* deletion mutant and wild-type (NCBI SRA database with accession numbers SRR12262932, SRR12262933, SRR12262934 (wild-type 1–3), and SRR12262935, SRR12262936, SRR12262937 (ΔCcPmk1-1–3)) (Table S5) [47] and found that only *CcbZIP05* and *CcbZIP23* were significantly down-regulated in the *CcPmk1*-deficient mutant compared to the wild-type (Figure 5A), which was also validated by using qRT-PCR (Figure 5B), indicating that they were modulated by *CcPmk1* and might be involved in fungal pathogenicity. Therefore, *CcbZIP05* and *CcbZIP23* were selected for further functional analyses.

In order to characterize the functions of *CcbZIP05* and *CcbZIP23* on fungal development and pathogenicity, we carried out gene knockout by homologous recombination. Briefly, the target bZIP gene in the wild-type was replaced by the hygromycin marker gene. Successful single integration deletion mutants were generated for both two *CcbZIP* genes (named Δ*CcbZIP05* and Δ*CcbZIP23*), and complementation of the two deletion mutants was performed respectively (named Δ*CcbZIP05-4/C* and Δ*CcbZIP23-1/C*) (Figure S3).

### 3.7. CcbZIP05 and CcbZIP23 Are Important for the Development of C. chrysosperma

**Figure 6:**
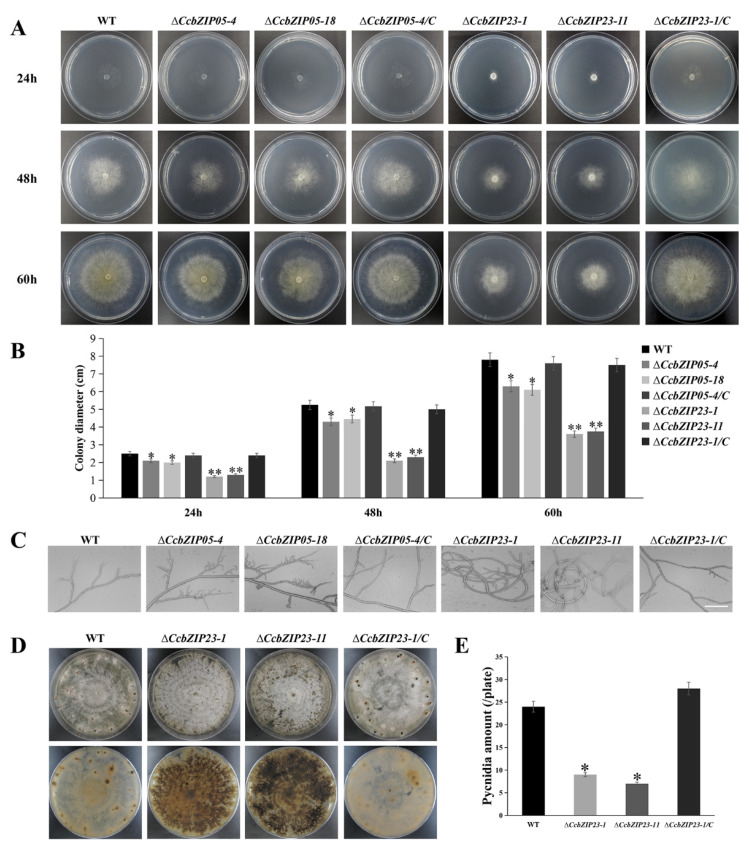
Phenotypic analyses of Δ*CcbZIP05* and Δ*CcbZIP23* mutants. (**A**) Colony morphologies of the wild-type, gene deletion mutants, and complemented strains after 24, 48, and 60 h grown on PDA plates. (**B**) Colony diameters of the strains on PDA plates shown in the part (**A**). (**C**) Hyphal branches in the tested strains cultured on PDA plates at 25 °C (scale bar = 50 μm). (**D**) Colony morphologies and pycnidia formation of the wild-type, gene deletion mutants, and complemented strains after 30 days growth on PDA plates. (**E**) Quantification of pycnidia production in each strain. The error bars represent the standard deviations based on three independent biological replicates. The asterisks indicate significant differences (* *p* < 0.05, ** *p* < 0.01).

To evaluate the functions of *CcbZIP05* and *CcbZIP23* on mycelia growth, the Δ*CcbZIP05* and Δ*CcbZIP23* deletion mutants were grown on PDA plates. Compared with the wild-type, the Δ*CcbZIP05* and Δ*CcbZIP23* mutants exhibited a significantly smaller colony diameter, and the growth of the complemented strains Δ*CcbZIP05-4*/C and Δ*CcbZIP23-1*/C on PDA media was restored to wild-type levels (Figure 6A,B). In addition, we observed the growth morphology of the mycelium under the light microscope. The results showed that the mycelia of the Δ*CcbZIP05* and Δ*CcbZIP23* mutants had more frequent branches than wild-type and complemented strains. In addition, the mycelial branch growth direction of Δ*CcbZIP23* mutants became twisted and coiled (Figure 6C). Interestingly, we found that Δ*CcbZIP23* but not Δ*CcbZIP05* mutants would accumulate obvious pigment in the PDA plates compared to the wild-type and complemented strains (Figure 6D, Figures S4 and S5).

Subsequently, we analyzed the conidial production of each strain. The result showed that Δ*CcbZIP23* mutants formed fewer pycnidia than wild-type and Δ*CcbZIP23-1*/C strains (Figure 6D,E), while Δ*CcbZIP05* mutants had no difference in the number of pycnidia compared with the wild-type (Figure S3). These results showed that *CcbZIP05* and *CcbZIP23* were involved in the fungal growth, and *CcbZIP23* regulated the conidial production and pigment formation in *C. chrysosperma*.

### 3.8. CcbZIP05 and CcbZIP23 Are Required for the Stress Responses

**Figure 7:**
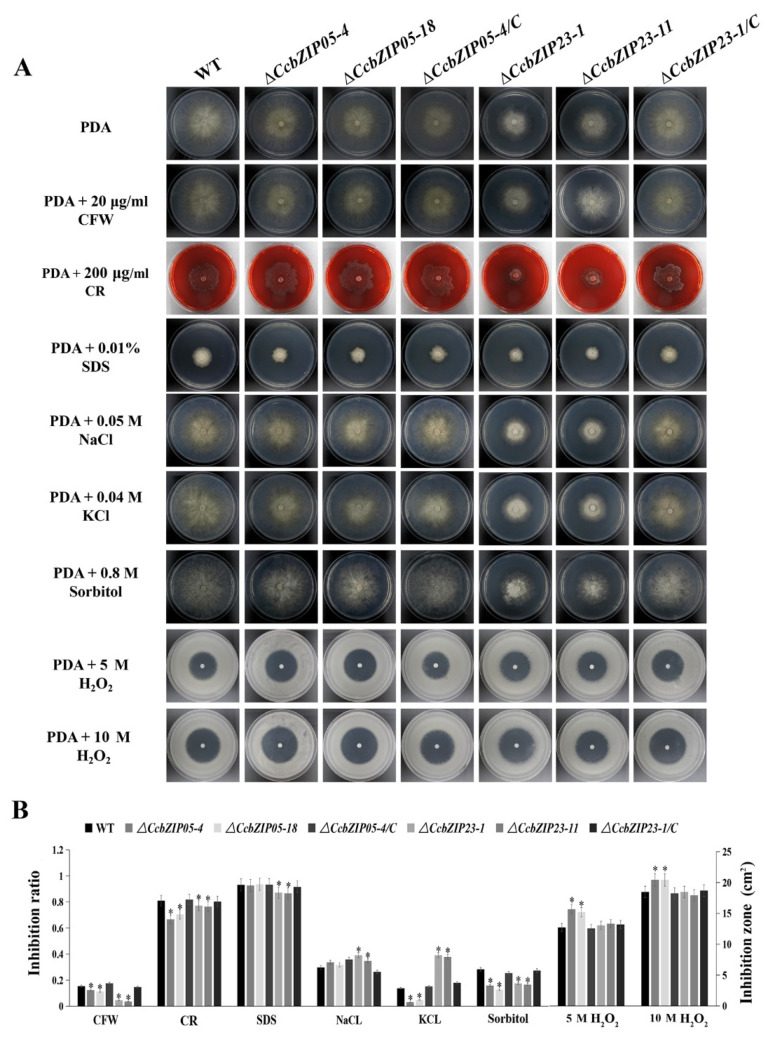
Involvement of Δ*CcbZIP05* and Δ*CcbZIP23* mutants in response to the abiotic stresses. (**A**) Comparison of the wild-type, gene deletion mutants, and complemented strains in colony morphology and stress tolerance. In oxidative stress test, conidial suspensions (1 × 10^5^ spores) of wild-type, gene deletion mutants, and complemented strain were spread on PDA plates. Sterile filter paper disks (5-mm diameter) were placed in the center of the plates, and 5 μL 5 M or 10 M H_2_O_2_ solution was added to each paper disk. The plates were incubated at 25 °C for 4 days, and the inhibition zones were observed and measured (**B**) The bar chart shows the inhibition rate or inhibition zone of the individual strains under different abiotic stresses shown in part (**A**). Error bars represent the standard deviations based on three independent replicates. Asterisks indicate significant differences at *p* < 0.05.

To evaluate the functions of *CcbZIP05* and *CcbZIP23* in cell wall integrity and stress responses, the fungal growths of the wild-type, Δ*CcbZIP05*/Δ*CcbZIP23*, and complemented strains on the PDA supplemented with cell wall interfering agents (CFW, CR, and SDS), osmotic interfering agents (NaCl, KCl, and sorbitol), and oxidative stress agent (H_2_O_2_) were analyzed. As shown in the Figure 7, the growth inhibition rate of the Δ*CcbZIP05* deletion mutants was significantly reduced on the PDA plate supplied with CFW, CR, and sorbitol compared with the wild-type, while no distinguished differences were found between the Δ*CcbZIP05* mutants and wild-type grown on the PDA plate supplemented with SDS, NaCl, and KCl. The data are summarized in Figure 7B. Additionally, the Δ*CcbZIP05* mutants were more sensitive to 5 M H_2_O_2_ or 10 M H_2_O_2_ stress than the wild-type and complemented strains (Figure 7B). Similarly, the growth of the Δ*CcbZIP23* mutants showed significantly lower growth inhibition than WT on PDA plate supplied with CFW, CR, SDS, and sorbitol, while the Δ*CcbZIP23* mutants enhanced their tolerance to salt stresses (NaCl and KCl) compared to the wild-type and complemented strains (Figure 7B). Intriguingly, Δ*CcbZIP23* mutants displayed comparable phenotypes compared to the wild-type and complemented strains on the PDA plates supplied with 5 M H_2_O_2_ or 10 M H_2_O_2_ (Figure 7B)_._ These results suggest that *CcbZIPs* are involved in stress responses, while they may show convergent and distinguished roles, etc.

**Figure 8:**
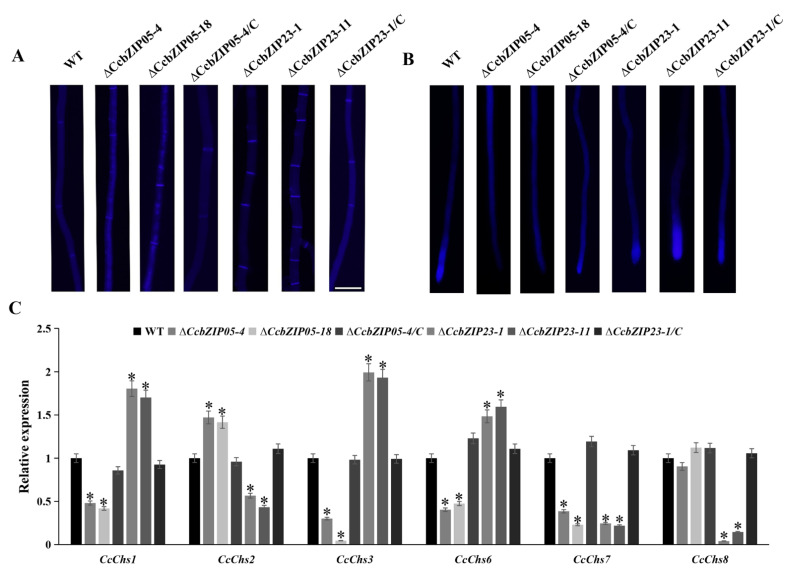
Chitin deposition and the expression of the putative chitin synthase-encoding genes in each strain. (**A**) Differences in the number of septa in mycelia. Bar = 50 μm. (**B**) Chitin deposition on hyphal tip. (**C**) Expression of chitin synthase-encoding genes in wild-type and deficient mutants. The qRT-PCR was used to measure the expression of chitin synthase-encoding genes in the wild-type, Δ*CcbZIP05* and Δ*CcbZIP23* mutants, and complemented strains. The *CcActin* gene was used as the reference gene. The experiments were repeated three times. The data were analyzed using one way ANOVA and Duncan’s range test. The asterisks indicate significant differences (*p* < 0.05).

Chitin is an essential component of the fungal cell wall, which plays important roles in hyphal growth and fungal morphogenesis. Therefore, we compared the chitin deposition among each strain by using the CFW staining. As shown in Figure 8A, B, obvious chitin deposition was observed at the septa and hyphal tips in the wild-type, Δ*CcbZIP23,* and complemented stains, while the chitin was not significantly accumulated at the hyphal tips of the Δ*CcbZIP05* mutants. Additionally, a significantly increased number of septa was found in Δ*CcbZIP23* mutants compared to the wild-type, Δ*CcbZIP05*, and complemented strains (Figure 8A). Subsequently, we calculated the expression of the putative chitin synthase-encoding genes (*CcChs1*, *CcChs2*, *CcChs3*, *CcChs6*, *CcChs7*, *CcChs8*), which had been analyzed in our previous works [48]. The results showed that the expression of *CcChs2* was significantly increased while the expression of *CcChs1*, *CcChs3*, *CcChs6*, and *CcChs7* was significantly reduced in the Δ*CcbZIP05* mutants (Figure 8C). As for the Δ*CcbZIP23* mutants, different expression patterns were found. The expression of *CcChs1*, *CcChs3*, and *CcChs6* was significantly up-regulated, while the expression of *CcChs2*, *CcChs7*, and *CcChs8* was significantly down-regulated (Figure 8C).

### 3.9. CcbZIP05 and CcbZIP23 Are Involved in Pathogenicity

**Figure 9:**
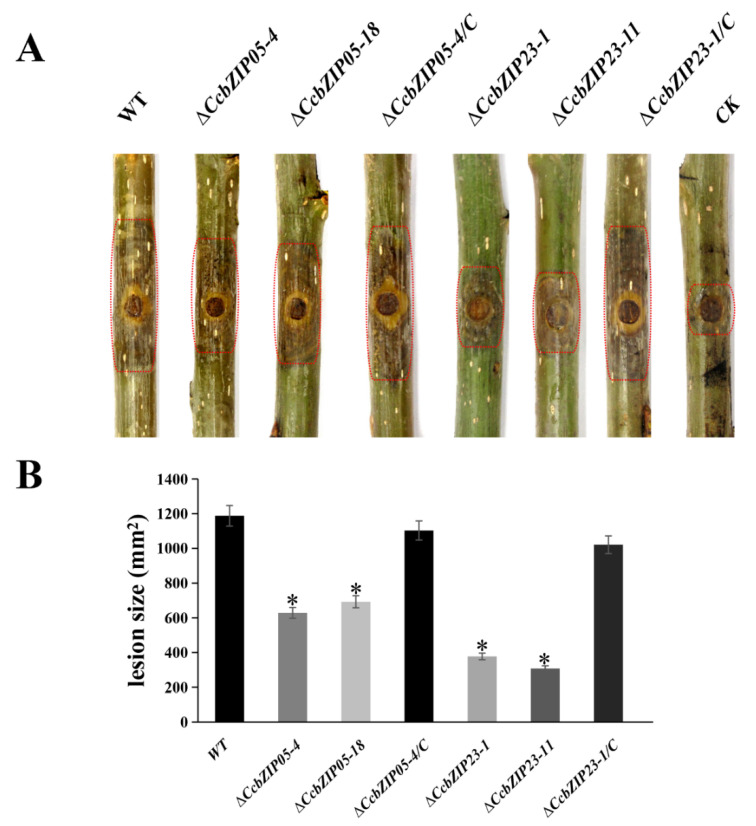
Pathogenicity test of Δ*CcbZIP05* and Δ*CcbZIP23* mutants on poplar twigs. (**A**) Infection symptoms on detached poplar twigs inoculated with the wild-type, gene deletion mutants, and complemented strains. The inoculated twigs were photographed at 6 dpi. CK represents twigs inoculated with PDA plugs after scalding. (**B**) Lesion areas were determined on the inoculated twigs. The asterisks on the bars indicate a significant difference between *CcbZIP05* deletion mutants, *CcbZIP23* deletion mutants, and the wild-type (*p* < 0.05). In each experiment, 20 healthy poplar twigs were inoculated with wild-type, gene deletion mutants, and complementary strains, respectively. The experiment was repeated three times.

To explore whether the functions of *CcbZIP0*5 and *CcbZIP23* are related to the pathogenicity of *C. chrysosperma*, we inoculated the wild-type, Δ*CcbZIP0*5 and Δ*CcbZIP23* mutants, and complemented strains on the poplar twigs. The lesion areas on poplar branches inoculated with the Δ*CcbZIP0*5 and Δ*CcbZIP23* mutants were significantly smaller than those inoculated with the wild-type and complemented strains at 6 dpi (Figure 9A,B). In consideration of the reduced growth rate of Δ*CcbZIP0*5 (29.9%) and Δ*CcbZIP23* (51.7%), the lesion areas on poplar branches inoculated with the Δ*CcbZIP0*5 and Δ*CcbZIP23* mutants were reduced by about 42.3% and 68.9% compared to the wild-type and complemented strains, respectively. Therefore, the results indicated that the reduced virulence of Δ*CcbZIP05* and Δ*CcbZIP23* mutants might partly be resulted from the defects in fungal growth.

### 3.10. CcbZIP05 and CcbZIP23 Differentially Regulate the Expression of Putative Effector Genes

**Figure 10:**
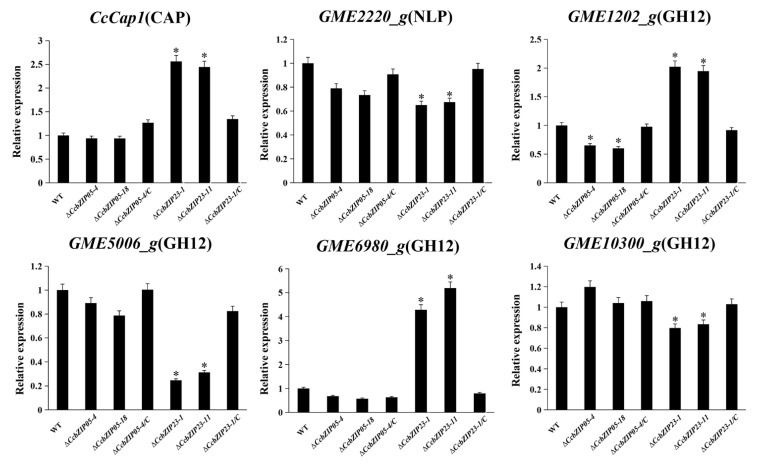
*CcbZIP05* and *CcbZIP23* differentially regulate expression of putative effector genes. The qRT-PCR was used to measure the expression levels of putative effector genes in the wild-type, Δ*CcbZIP05* and Δ*CcbZIP23* mutants, and complemented strains. The *CcActin* gene was used as the reference gene. The experiments were repeated three times. The data were analyzed using one-way ANOVA and Duncan’s range test. The asterisks indicate significant differences (*p* < 0.05).

Transcription factors can regulate the expression of downstream targets to activate or suppress their functions. Our previous studies found that the *CcPmk1* and the Gti1/Pac2 transcription factor *CcSge1* could regulate the expression of putative effector genes [48,49]. Therefore, we calculated the expression of putative effector genes in Δ*CcbZIP05* and Δ*CcbZIP23* mutants including the *CcCAP1* (Cysteine-rich secretory proteins, Antigen 5, and Pathogenesis-related 1 protein), glycoside hydrolase genes (*GME1202_g*, *GME5006_g*, *GME6980_g*, *GME10300_g*), and Nepl-like gene (NLP *GME2220_g*). As shown in Figure 10, the expression of *GME2220_g*, *GME5006_g,* and *GME10300_g* was significantly decreased in Δ*CcbZIP23* mutants, but they showed comparable expression level among wild-type, Δ*CcbZIP05* mutants, and complemented strains. However, the expression of *CcCAP1* and *GME6980_g* was significantly up-regulated in the Δ*CcbZIP23* mutants compared to the wild-type. Intriguingly, a different expression pattern of *GME1202_g* was found in Δ*CcbZIP05* and Δ*CcbZIP23* mutants, which was significantly down-regulated in the Δ*CcbZIP05* mutants but significantly up-regulated in the Δ*CcbZIP23* mutants (Figure 10).

## 4. Discussion

Transcription factors (TFs) regulate the expression of downstream genes and are involved in a variety of key cellular functions. They are considered to be important components of the signal transduction pathway and are the crucial link between signal flow and the expression of the target gene. The bZIP TFs are one of the largest and the most diverse TF families, which are widely and exclusively distributed in eukaryotes. Many key biological processes require bZIP TFs, such as various stress responses, fungal growth, primary and secondary metabolism, and pathogenicity of phytopathogenic fungi [9,46,63]. However, no available data of bZIP TFs in *C. chrysosperma* were reported. Hence, in this study, we systematically identified a comprehensive set of 26 bZIP TF members in the *C. chrysosperma* genome, and functionally characterized the roles of *CcbZIP05* and *CcbZIP23* in the fungal growth, stress responses, and pathogenicity, which will provide insights for functional research of other bZIP genes in *C. chrysosperma*.

Since the lifestyle of fungi heavily depends on their adaptations to their environment, replicated genes may lead to new functions and improve adaptations in a changing environment. In pathogenic fungi, improved nutrient uptake and more efficient catabolism, resistance, and adaptation to host infection can also be obtained through the expansion of gene families [64]. The bZIP superfamily is reported to have evolutionarily evolved from a single eukaryotic gene ancestor and has experienced multiple independent expansions [60]. Phylogenetic analysis of *CcbZIPs* and other selected fungal bZIP TFs showed that they were evenly distributed in eight clades (A–H), suggesting the bZIP gene family was present when these fungi were undifferentiated. Only the leucine zipper region of *CcbZIP23* and *CcbZIP26* lacked the following heptad repeats of the leucine (L), and the core arginine (R) had a phase 1 intron (Figure 1 and Figure 3). In eukaryotes, the evolution of introns may have a role in the functional evolution of paralogs, and more introns usually imply more complex regulation [58,59]. Genetic structure analysis revealed differences in the number of introns in *CcbZIPs* (Figure 2). The *CcbZIP* genes contained 0 to 3 introns, but the maximum number of introns was lower than that of *C. minitans* (max. six introns) and *U. virens* (max. four introns). It has been shown that introns are lost faster than they are gained after segmental replication [65], indicating a putative gene duplication event happened in a recent period in *C. chrysosperma* compared to the *C. minitans* and *U. virens*. In addition, eight bZIP domains were inserted by introns in the N-X_7_-R region. Similar results were found in the bZIP domains of *C. minitans* and *U. virens* [23,25].

Previous studies showed that some *bZIP* genes were required for host infection in *M. oryzae,* and they also exhibited increased expression levels in the infection stages, such as *MoATF1*, *MoHAC1*, *MoAP1*, *MoBZIP10*, and *MoMETR* [22,38]. According to our expression profile data, 15 *CcbZIP* genes showed significant changes at the early infection stages, indicating the involvement of these genes in pathogenicity (Figure 4). Additionally, some of their homologous genes are involved in fungal virulence. For example, overexpression of *meaB* (*CcbZIP02* homologue) can reduce the production of aflatoxin B1 and thus attenuate the virulence of *Aspergillus flavus* [66]. *VdAtf1* (*CcbZIP03* homologue) is involved in the virulence of *V. dahliae* by mediating nitrogen metabolism [67]. *VdHapX* (*CcbZIP07* homologue) controls iron homeostasis and is also crucial for the virulence of *V. dahliae* [68]. *AaMetR* (*CcbZIP20* homologue, a methionine biosynthesis regulator) contributes to virulence and oxidative stress tolerance in *Alternaria alternata* [69]. However, the *CcbZIP* genes that did not differentially express during the initial infection stages might also contribute to the fungal virulence. For example, the *CcbZIP25* homolog gene *Yap1* is required for fungal virulence and stress response in *C. gloeosporioides* and *M. oryzae* [70,71], but the *Bap1* (*Yap1* homolog) has no impact on pathogenicity and differentiation in *B. cinerea* [30]. In this study, *CcbZIP05* and *CcbZIP23* were involved in the fungal virulence of *C. chrysosperma*. The *ΔCcbZIP05* and *ΔCcbZIP23* mutants showed significantly reduced lesion sizes on poplar twigs compared to the wild-type. However, previous studies showed that deletion of *VDAG_0864*0 (*CcbZIP05* homologue) in *V. dahliae* showed no significant defects in fungal growth, stress responses, pathogenicity, and microsclerotia formation [72]. Moreover, similar results were found in *MGG_07305* (*CcbZIP05* homologue) of *M. oryzae* [22]. Additionally, deletion of *MGG_00587* (*CcbZIP23* homologue) of *M. oryzae* also did not affect fungal growth, appressorium formation, and fungal pathogenicity, while significantly increased conidiation was observed in the *MGG_07305* deletion mutants [38]. Here, we found that deletion of *CcbZIP23* significantly compromised the conidiation. The results suggest that the bZIP orthologs in different fungal species may display distinct functions.

Pmk1 is a pathogenicity-related component of the MAPK signaling pathway in almost all the pathogenic fungi and plays crucial roles in fungal development, pathogenicity, and stress response through the regulation of other genes [48]. In *Schizosaccharomyces pombe*, the Pmk1 MAPK pathway is involved in cell wall integrity through regulating the Atf1 expression [73]. In *M. oryzae*, a *bZIP* gene, *MGG_00587, was* significantly down-regulated during early appressorium development (4 h) in the Δpmk1 mutant compared to wild-type [74]. In *C. chrysosperma*, *CcbZIP05 and CcbZIP23* were significantly down-regulated in Δ*CcPmk1* (Figure 5). Therefore, it is considered that *Pmk1* can regulate the expression of downstream TF genes, including the bZIP TFs. Here, we found that the Δ*CcbZIP05* and Δ*CcbZIP23* mutants showed some similar phenotypes as the *ΔCcPmk1* deletion mutant, such as the reduced fungal growth, conidiation, stress response, and increased branching. On the other hand, some different defects were observed in Δ*CcbZIP23* mutants compared to the Δ*CcPmk1* mutants. For example, *CcbZIP23* deletion mutants accumulated obvious pigment in the plates, increased resistance to the CFW, CR, and sorbitol stresses, and reduced fungal virulence while the Δ*CcPmk1* mutants were nonpathogenic [48]. These results suggest that *CcbZIP05* and *CcbZIP23* may also be regulated by other components in addition to *CcPmk1*.

Fungal secondary metabolites are essential in the competition, defense, and development of fungi [75,76]. Transcriptional regulation plays an important role in the biosynthesis of fungal secondary metabolites [77]. In *Aspergillus flavus*, the bZIP TF *AflRsmA* regulates Aflatoxin B_1_ (AFB_1_, a potent carcinogen) biosynthesis through the oxidative stress pathway. Overexpression of *AflrsmA* increases AFB_1_ production [46]. Similarly, the deletion of *Afap1* (a bZIP TF) significantly reduces the production of AFB_1_ [78]. In *Aspergillus nidulans*, activation of *RsmA* (a YAP-like bZIP gene) greatly increases the production of secondary metabolites through binding to the two sites of the *AflR* promoter region (a C6 TF involved in the production of the carcinogenic and anti-predation secondary metabolites, namely, sterigmatocystin) [79]. In this study, deletion of *CcbZIP23* significantly increased the pigmentation accumulation in the plates (Figure 6 and Figure S5). These results indicate that *CcbZIP23* is involved in the secondary metabolism in *C. chrysosperma*, but the regulatory mechanism remains to be investigated.

Previous studies have shown that the fungal cell wall is composed mainly of chitin and glucan. Chitin synthesis is mainly mediated by chitin synthase. In plant pathogenic fungi, there are generally seven or eight chitin synthase-encoding genes [80,81]. In *M. oryzae*, three *CHS* genes (*CHS1*, *CHS2,* and *CHS6*) are involved in conidiation, pathogenicity, and stress responses [80]. In *F. graminearum*, Δ*FgChs2* and Δ*FgChs5* mutants (*CHS2*, *CHS6* homologous gene, respectively) show significantly reduced mycelial growth and conidiation, but they show increased sensitivity to CR [81]. In *Neurospora crassa*, the *chs-1^RIP^* mutant displays abnormal branching, swollen hyphal tips, and reduced growth rate; in transformants expressing *CHS3-GFP* or *CHS6-GFP* constructs, GFP signals are detected mainly at the hyphal tip, suggesting that they may be involved in polarized growth [82]. In our study, we found that the expression of chitin synthase-encoding genes was differentially regulated by *CcbZIP05* and *CcbZIP23*, which may partly contribute to the defects in chitin distribution, fungal growth, conidiation, stress responses, and pathogenicity in Δ*CcbZIP05* and Δ*CcbZIP23* mutants.

It is well known that pathogens will deliver numerous effectors into plant cells or the apoplast to suppress host immunity and then promote the infection or colonization [83]. Many reports showed that the TFs could regulate the expression of downstream effector genes [84,85]. In this study, we calculated the expression of several putative effector genes in Δ*CcbZIP05* and Δ*CcbZIP23* mutants, and their homologues were found to be involved in the fungal pathogenicity such as the glycoside hydrolase family 12 [86], Nep1-like proteins [87,88]. We found that *CcbZIP05* and *CcbZIP23* could regulate the expression of glycoside hydrolase 12 family genes, NLP gene, and CcCAP1, which may partly contribute to fungal virulence. In conclusion, we identified a total of 26 bZIP TF family members in the *C. chrysosperma* genome and analyzed the functions of *CcbZIP05* and *CcbZIP23*, which played important roles in fungal development, stress responses, and virulence of *C. chrysosperma*. The results provide a comprehensive view of the CcbZIP TF family and provide a basis for further studies on the function of other *bZIP* genes.

## Figures and Tables

**Table 1:** Genes encoding CcbZIP transcription factor.

CcbZIP Number	Locus Number	gDNA(bp)	cDNA(bp)	Intron	Protein Length (aa)	bZIP Domain Position	MW (kDa)	pI	Location on Supercontig
*Ccbzip01*	*GME965_g*	2143	930	2	309	132–199	32.57	6.097	Scaffold1:3424294:3426436: −
*Ccbzip02*	*GME1427_g*	1444	1224	2	407	65–127	43.65	5.919	Scaffold1:5014645:5016088: −
*Ccbzip03*	*GME1822_g*	1723	1551	2	516	401–466	54.55	6.532	Scaffold1:6461072:6462794: +
*Ccbzip04*	*GME2112_g*	2393	684	2	244	30–91	26.88	8.302	Scaffold1:7520918:7523310: +
*Ccbzip05*	*GME2174_g*	800	632	2	210	101–165	23.16	5.537	Scaffold1:7806590:7807389: +
*Ccbzip06*	*GME4009_g*	1081	1029	1	342	162–233	37.89	5.715	Scaffold4:2286665:2287745: −
*Ccbzip07*	*GME4152_g*	1881	1881	0	626	40–108	66.21	7.38	Scaffold4:2835713:2837593: −
*Ccbzip08*	*GME4174_g*	569	492	1	163	41–105	18.24	5.664	Scaffold4:2902921:2903489: +
*Ccbzip09*	*GME4540_g*	1044	1044	0	347	232–296	37.37	6.81	Scaffold7:356347:357390: +
*Ccbzip10*	*GME4781_g*	1738	1389	3	462	76–137	49.77	7.161	Scaffold3:232131:233868: +
*Ccbzip11*	*GME4894_g*	1953	1893	1	630	259–323	69.87	4.991	Scaffold3:653712:655664: +
*Ccbzip12*	*GME5553_g*	1038	735	1	227	54–113	24.96	4.635	Scaffold3:2936931:2937968: −
*Ccbzip13*	*GME6051_g*	1884	924	2	307	234–299	33.9	4.825	Scaffold5:131906:133789: +
*Ccbzip14*	*GME6598_g*	1800	1800	0	599	349–412	63.93	11.082	Scaffold5:2160568:2162367: −
*Ccbzip15*	*GME7181_g*	1654	1509	2	502	94–158	54.69	8.404	Scaffold6:723065:724718: −
*Ccbzip16*	*GME7284_g*	1281	963	2	320	133–201	35.05	5.258	Scaffold6:1084879:1086159: +
*Ccbzip17*	*GME8123_g*	771	771	0	256	160–221	28.21	5.995	Scaffold2:1195066:1195836: +
*Ccbzip18*	*GME8194_g*	1866	1794	1	597	293–358	65.51	4.698	Scaffold2:1441851:1443716: +
*Ccbzip19*	*GME8362_g*	1835	1683	1	560	85–151	61.53	6.693	Scaffold2:2031527:2033361: +
*Ccbzip20*	*GME8516_g*	1635	852	1	283	174–235	31.53	5.486	Scaffold2:2636438:2638072: −
*Ccbzip21*	*GME9378_g*	1755	1755	0	584	448–514	64.68	6.148	Scaffold2:5864980:5866734: −
*Ccbzip22*	*GME9737_g*	1192	1041	2	346	294–341	37.37	5.741	Scaffold2:7266318:7267509: −
*Ccbzip23*	*GME9746_g*	1149	858	2	285	63–108	31.3	7.702	Scaffold2:7310294:7311442: −
*Ccbzip24*	*GME9819_g*	2194	1902	2	633	79–138	69.52	5.308	Scaffold2:7606002:7608197: +
*Ccbzip25*	*GME9912_g*	2022	1800	3	599	149–214	64.89	4.863	Scaffold2:7925885:7927906: +
*Ccbzip26*	*GME10373_g*	1438	960	3	319	8–61	35.7	6.338	Scaffold2:9540692:9542129: −

## Data Availability

Data is contained within the article or Supplementary Material.

## Supplementary Materials

The following are available online at https://www.mdpi.com/article/10.3390/jof8010034/s1. Figure S1: Distributions of conserved domains in CcbZIP genes. All CcbZIP genes manually confirmed the existence of domains by using the InterProScan on EBI web server (http://www.ebi.ac.uk/interpro/, accessed on 26 December 2019). Different domains are highlighted in different color. Figure S2: Phylogenetic analysis of bZIP members from nine fungi. The maximum likelihood trees were constructed based on 181 full-length protein sequences. The phylogenetic tree was constructed using raxmlGUI1.3 software and the bootstrap test carried out with 1000 iterations. The bZIP members are clustered into eight clades (A–H) indicated by colored branches. The CcbZIPs are denoted by red circles. The sequences were collected from organisms as follows: Cc, Cytospora chrysosperma; Bc, Botrytis cinerea; Cg, Colletotrichum gloeosporioides; Cp, Cryphonectria parasitic; Fg, Fusarium graminearum; Mo, Magnaporthe oryzae; Sc, Saccharomyces cerevisiae; Ss, Sclerotinia sclerotiorum; Vd, Verticillium dahliae. Figure S3: Construction and confirmation of CcbZIP05 and CcbZIP23 disrupted and complemented mutants. (A) The abridged general view of the generation of deletion mutants for the CcbZIP05 gene. (B) The abridged general view of the generation of deletion mutants for the CcbZIP23 gene. Southern blot analysis was conducted to determine the single integration events, which are indicated on each schematic map. Figure S4: Colony morphologies and pycnidia formation. (A) The colony morphologies and pycnidia formation of the wild-type, ΔCcbZIP05 mutants, and complemented strains after 30 days of growth on PDA plates. (B) Quantification of pycnidia production in each strain. Figure S5: Colony morphologies of wild-type, ΔCcbZIP23 mutants, and complemented strain grown on the PDA supplemented with cell wall interfering agents (CFW and SDS) and osmotic interfering agents (NaCl, KCl, and sorbitol) at 30 days. Table S1: Primers used in this study. Table S2: Sequence information of bZIP genes in this study. Table S3: Homologous genes of CcbZIPs in the PHI database. Table S4: The expression of CcbZIPs at different time of infection. Table S5: Expression of CcbZIP transcription factor genes in wild-type and CcPmk1-deficient mutant.
