# Supplementary figures and images for: Genome-Wide Identification of bZIP Transcription Factor Genes and Functional Analyses of Two Members in Cytospora chrysosperma
Source: J Fungi (Basel). 2021 Dec 30;8(1):34. doi: 10.3390/jof8010034 (PMC8778692; doi:10.3390/jof8010034)

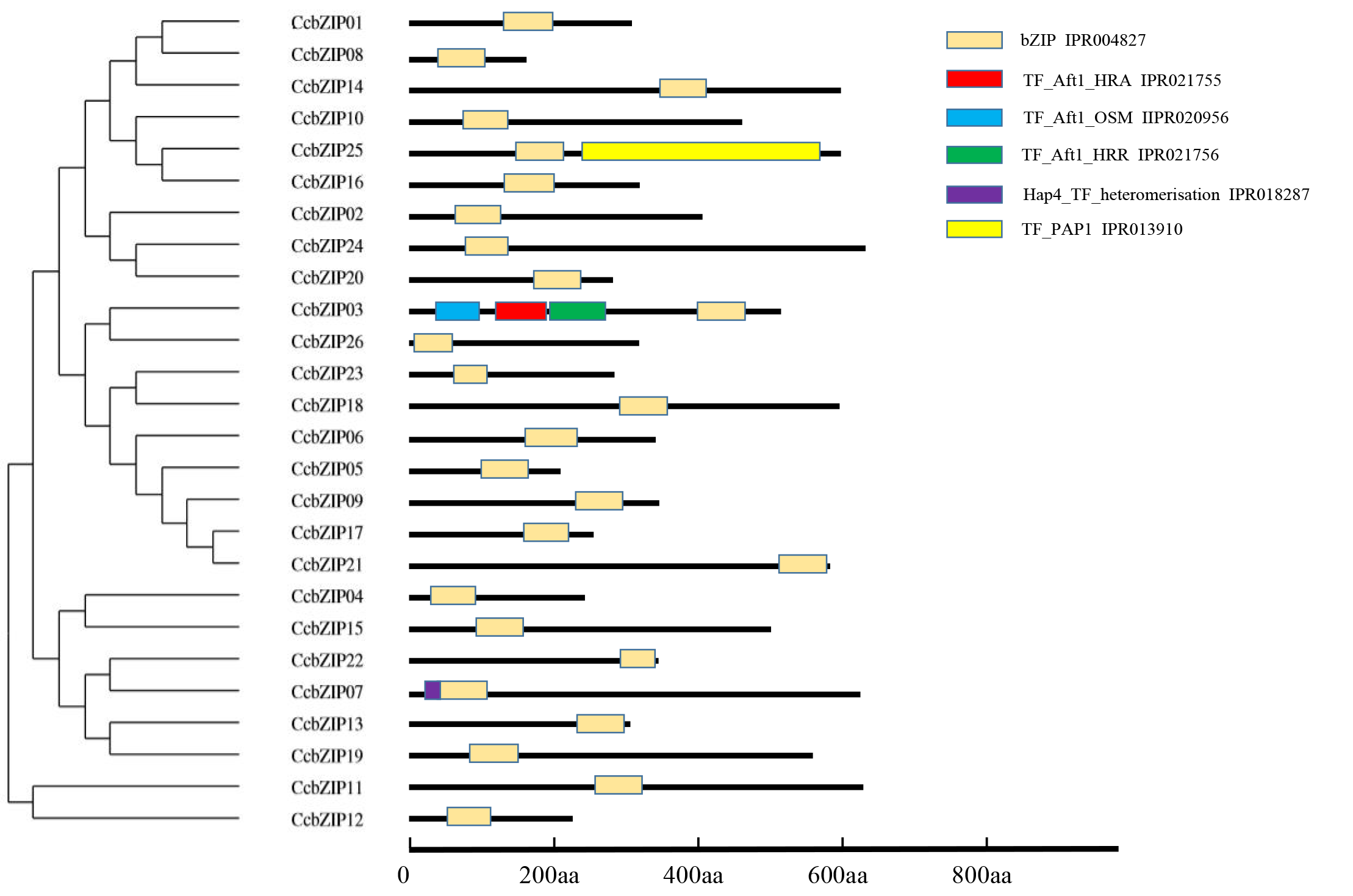

Supplement: Supplementary file 1 [file jof-08-00034-s001.zip › Figure S1.tif]

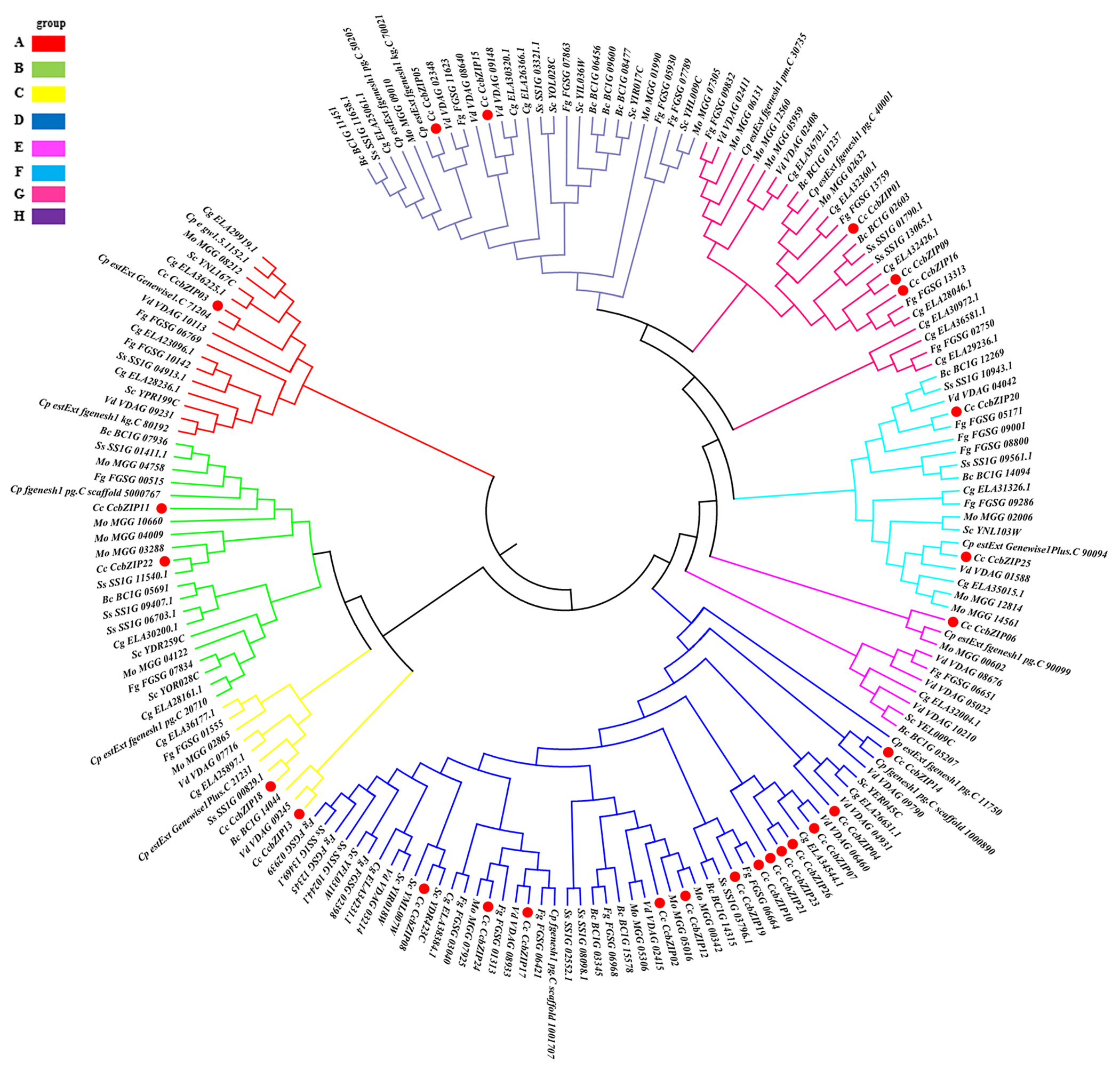

Supplement: Supplementary file 1 [file jof-08-00034-s001.zip › Figure S2.tif]

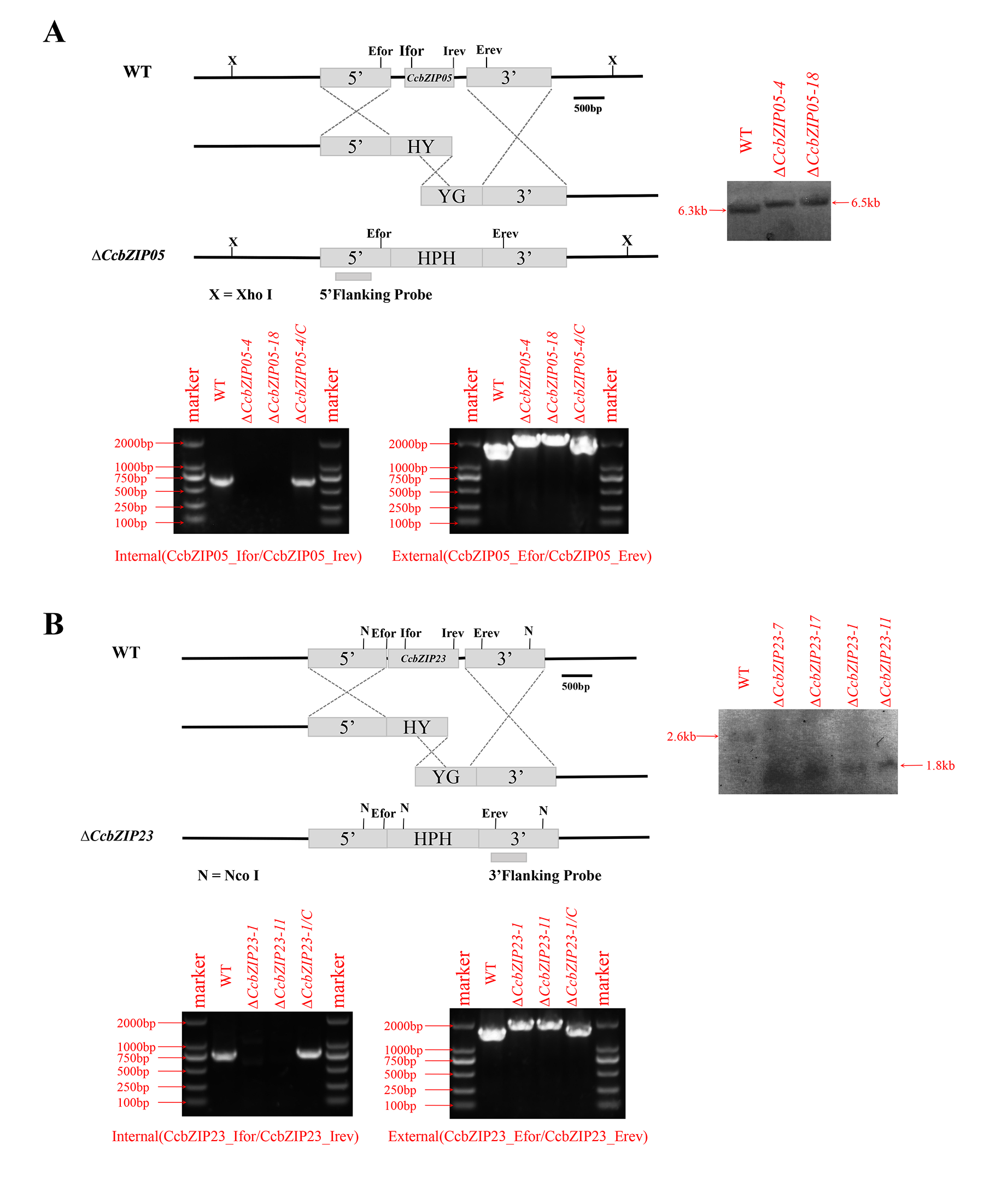

Supplement: Supplementary file 1 [file jof-08-00034-s001.zip › Figure S3.tif]

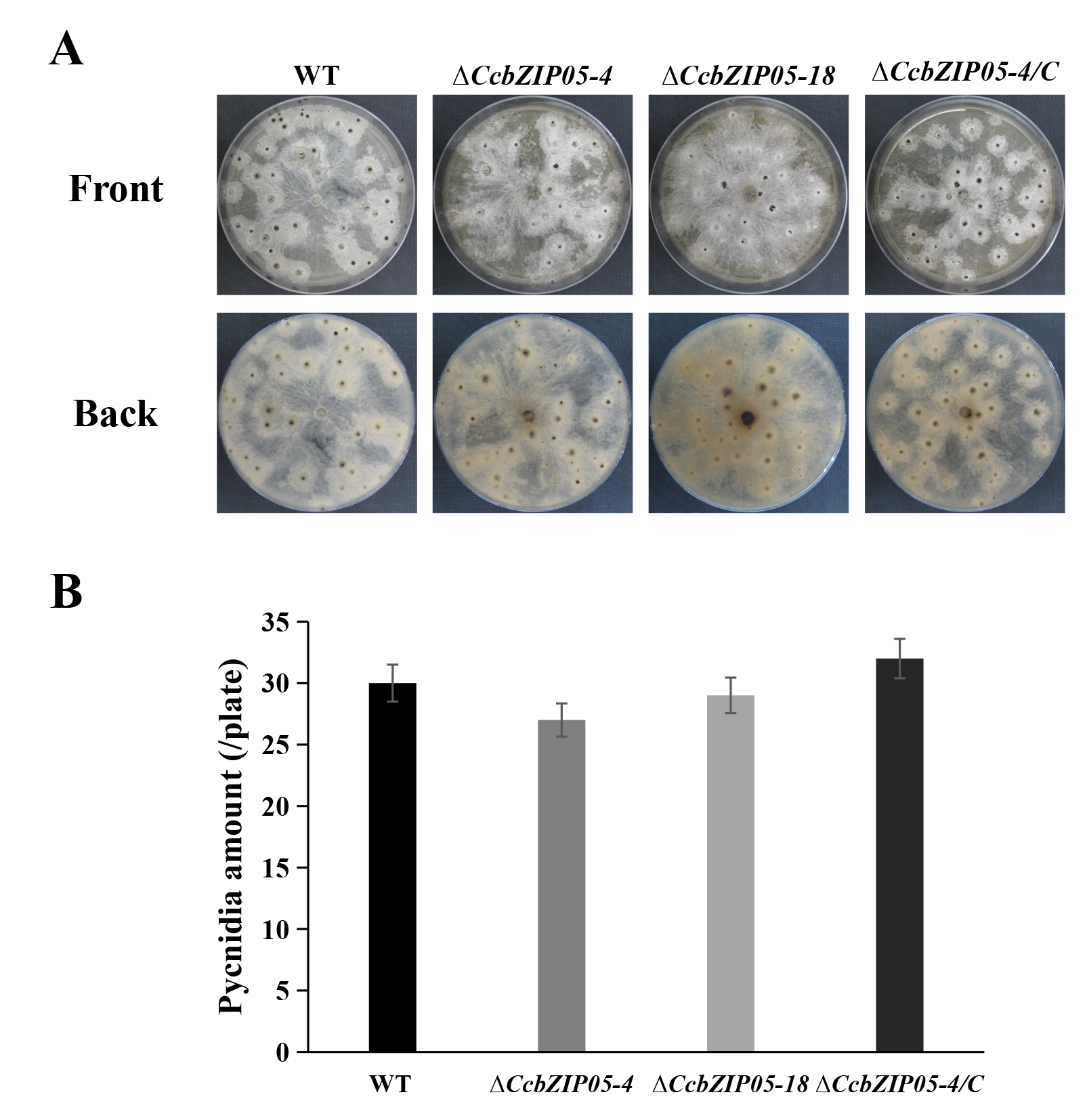

Supplement: Supplementary file 1 [file jof-08-00034-s001.zip › Figure S4.tif]

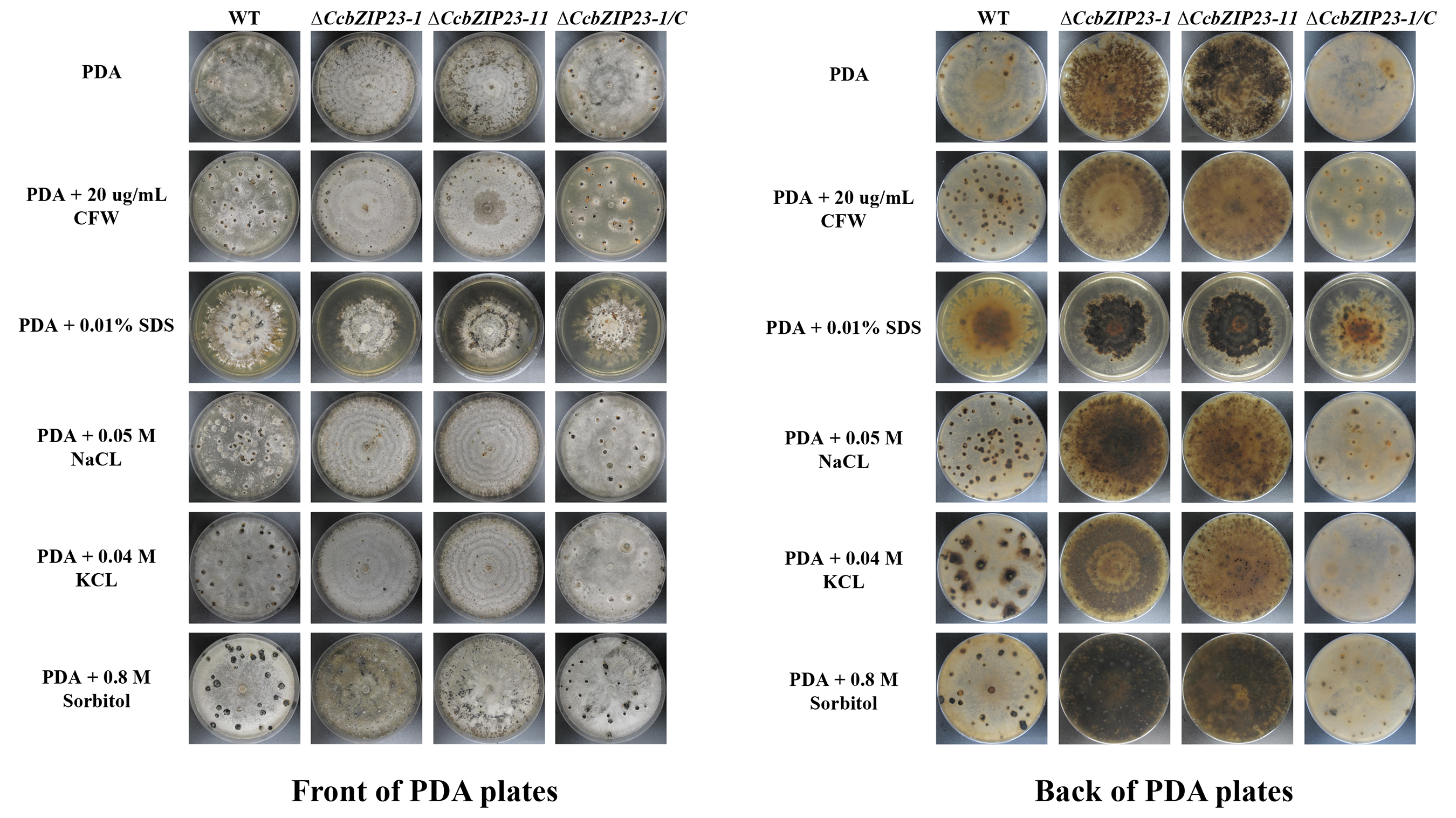

Supplement: Supplementary file 1 [file jof-08-00034-s001.zip › Figure S5.tif]
